# Improvisation and live accompaniment increase motor response and reward during a music playing task

**DOI:** 10.1038/s41598-024-62794-6

**Published:** 2024-06-07

**Authors:** Anna Palumbo, Karleigh Groves, Eva Luna Munoz-Vidal, Alan Turry, Robert Codio, Preeti Raghavan, Heidi Schambra, Gerald T. Voelbel, Pablo Ripollés

**Affiliations:** 1 Rehabilitation Sciences Program, Steinhardt School of Culture, Education, and Human Development, New York University, New York, NY 10003 USA; 2 Department of Psychology, New York University, New York, NY 10003 USA; 3 Music and Audio Research Lab, New York University, New York, NY 10003 USA; 4 Center for Language, Music, and Emotion (CLaME), New York University, New York, NY 10003 USA; 5 Department of Music and Performing Arts Professions, Steinhardt School of Culture, Education, and Human Development, New York University, New York, NY 10003 USA; 6 Nordoff-Robbins Center for Music Therapy, New York University, New York, NY 10003 USA; 7 Department of Physical Medicine and Rehabilitation and Neurology, Johns Hopkins University School of Medicine, Baltimore, MD 21287 USA; 8 New York University Grossman School of Medicine, New York, NY 10016 USA; 9 Department of Occupational Therapy, Steinhardt School of Culture, Education, and Human Development, New York University, New York, NY 10003 USA; 10 Center of Health and Rehabilitation Research, New York University, New York, NY 10003 USA; 11 Department of Rehabilitation Medicine, Rusk Rehabilitation, NYU Langone Health, New York, NY 10016 USA

**Keywords:** Physiology, Psychology, Motor control, Reward

## Abstract

Music provides a reward that can enhance learning and motivation in humans. While music is often combined with exercise to improve performance and upregulate mood, the relationship between music-induced reward and motor output is poorly understood. Here, we study music reward and motor output at the same time by capitalizing on music playing. Specifically, we investigate the effects of music improvisation and live accompaniment on motor, autonomic, and affective responses. Thirty adults performed a drumming task while (i) improvising or maintaining the beat and (ii) with live or recorded accompaniment. Motor response was characterized by acceleration of hand movements (accelerometry), wrist flexor and extensor muscle activation (electromyography), and the drum strike count (i.e., the number of drum strikes played). Autonomic arousal was measured by tonic response of electrodermal activity (EDA) and heart rate (HR). Affective responses were measured by a 12-item Likert scale. The combination of improvisation and live accompaniment, as compared to all other conditions, significantly increased acceleration of hand movements and muscle activation, as well as participant reports of reward during music playing. Improvisation, regardless of type of accompaniment, increased the drum strike count and autonomic arousal (including tonic EDA responses and several measures of HR), as well as participant reports of challenge. Importantly, increased motor response was associated with increased reward ratings during music improvisation, but not while participants were maintaining the beat. The increased motor responses achieved with improvisation and live accompaniment have important implications for enhancing dose of movement during exercise and physical rehabilitation.

## Introduction

Music provides a pleasurable and motivating stimulus that is associated with activity in reward- and emotion-related brain regions^1^. Further, peak moments of musical pleasure are associated with dopaminergic release^2,3^. The rewarding nature of music is thought to derive, at least in part, from our ability to learn its structure and make predictions about how it will progress^4–7^, although autobiographical memories^8–10^ and individual traits^11–13^, among others, also contribute to music reward. Importantly, the experience of groove, or the pleasurable motivation to move to music, also activates reward and motor-related brain areas^14^. The dual activation of reward and motor-related brain regions by music make it an ideal stimulus to examine the relationship between movement and reward.

Reward plays an important role in motor performance and learning^15^. In non-human animals, dopaminergic signaling between reward and motor-related brain areas optimizes motor skill learning^16,17^. In humans, monetary reward during motor tasks increases force production, reduces reaction time, improves recall of motor skills, increases neural activation in reward-related areas and modulates corticospinal excitability^18–20^. Similarly, music is frequently combined with exercise and athletic training as a rewarding stimulus to improve mood and performance^21,22^. Listening to music improves affect, endurance, speed, power output, movement kinematics, and cardiovascular efficiency^23–29^. As a readily accessible stimulus, music provides an important reward mechanism to examine in relationship to motor performance.

Capitalizing on the rewarding nature of music, motor rehabilitation interventions have integrated the use of music playing (i.e., using musical instruments) to improve recovery following acquired brain injury, leading to increased motor function, reduced depression and improved positive affect, and increased activation in the affected sensorimotor cortex^30–34^. Critically, music playing supports higher levels of motor recovery among individuals with greater sensitivity to musical reward^35^. This evidence suggests that increasing reward during music playing improves motor outcomes. However, the mechanisms supporting these effects remain unclear. Here we capitalize on music playing to investigate the link between movement and reward. We examine two avenues for enhancing reward and motor output during music playing: music improvisation and live accompaniment.

Music improvisation involves creating or adapting music in the moment, providing choice about what to play^36,37^. Choice enhances motor performance and learning, even when the choice is unrelated to the task, and increases motivation and activation in reward-related brain areas^38–42^. In this vein, choice in combining sounds increases enjoyment and interest^43^. Music improvisation, compared to playing a pre-defined set of notes, increases activation in the sensorimotor cortex and in brain regions related to executive functions^5,44,45^. Creativity in music improvisation correlates with grey matter in the hippocampus^46^. These findings indicate the potential for music improvisation to influence cognition (e.g., attention, learning), motor responses, and reward during music-playing.

Live accompaniment creates a social experience during music playing. Positive social interactions contribute to reward^47^, and cooperative learning of motor tasks increases intrinsic motivation, positive affect, self-efficacy, and motor learning as compared to competitive or non-interactive learning^48^. Live music also modulates heart rate and mood, including self-reports of reduced anxiety and improved vigor (mood states which are distinct from reward, but have associations with reward processing^49,50^) as compared to recorded music^51,52^. Although recorded music has been demonstrated to produce levels of pleasure (i.e., the ‘liking’ or hedonic component of reward^53^) similar to live music during listening^54^, collective experience in music (i.e., moving together during music) has been linked to increased movement energy^55^. Similarly, greater movement synchrony is associated with increased emotional expression during music playing^56^ and increased social affiliation during finger tapping^57^. Together, these findings indicate that social interactions during live accompaniment may enhance reward and motor activation during music playing.

Here, we compare the effects of improvisation and live accompaniment in a music-playing task. Participants played a drum while either (i) improvising or maintaining the beat and (ii) playing with live or recorded accompaniment. We designed our study to be relevant to clinical use of music (i.e., stroke rehabilitation^33,58^), with a music therapist providing the accompaniment, both by creating the recordings and playing live. The music therapist played responsively with the participant during the improvisation condition with live accompaniment (i.e., imitating rhythmic patterns or dynamics), to better reflect the clinical dynamics during music therapy^36,59^.

To investigate the link between reward and movement, we measured affective, motor, and autonomic responses to the music playing tasks. Affective responses were measured by behavioral self-reports after each music-playing trial. Motor responses were measured using electromyography (EMG), accelerometry, and the drum strike count. Whereas EMG assessed peak muscle activation at each movement, accelerometry assessed total activity during each playing task. The drum strike count provided an objective measure of how often participants engaged in music playing for each task, which can be independent of the force applied while playing. Autonomic responses were measured using electrodermal activity (EDA) and electrocardiogram (ECG), for which we assessed heart beats per minute (BPM) and heart rate variability (HRV). EDA is frequently used in studies assessing reward responses to music^60^ but is susceptible to motion artifacts that may arise during music playing. We therefore collected ECG as an additional measure of physiological arousal to ensure adequate measurement of autonomic responses. We predicted that improvisation combined with live accompaniment would produce the highest levels of motor activity, positive affect, and autonomic arousal, as measured across all outcomes. We also predicted that increased motor responses would correlate with increased self-reports of reward-related affective responses during music-playing, thus supporting the link between reward and motor performance.

## Methods

### Participants

Thirty healthy adults were recruited from the New York University community, ages 18–58 (M = 22.9, SD = 8.11; 19 female, 10 male, 1 non-binary). Sample size was based on previous studies with 13–21 participants demonstrating that rhythmic improvisation increases the number of notes played and the activation of motor planning and executive brain areas^45,61^. All participants were either enrolled in university (n = 24) or had already earned an undergraduate or graduate degree or other professional qualification and were currently employed (n = 4). Participants self-reported their race as Asian (n = 14), White/Caucasian (n = 8), Black/African American (n = 2), or Mixed (n = 2), with 3 participants not reporting their race. Twenty seven percent (n = 8) of participants reported Hispanic ethnicity. Inclusion criteria included right-handedness with no history of brain injury. Exclusion criteria included musical anhedonia, as indicated by a score of < 65 on the Barcelona Music Reward Questionnaire^11^, or current employment as a professional musician. The participants gave written informed consent as per the institutional review board at New York University and were paid or received university course credit for their participation.

### Music playing task

Participants played a drum along with four one-minute piano compositions, written specifically for this study (see Audio S1, S2, S3, and S4 in Supplementary Information for recordings of each composition). Each of the four piano compositions were based on one of the following musical styles: March, Waltz, Salsa, and Rock. The musical styles were selected to reflect the variety used in real world music-playing interventions. In so doing, we aimed to support the generalizability of our findings for clinical music interventions. However, to account for potential variability due to piano composition style, we control for this in all analyses by adding piano composition style as a fixed factor in our models. In describing how participants were instructed to play with the piano compositions, we refer to both the beat and rhythm of the music. We define the beat as the pulse of the music while the rhythm is the pattern of temporal intervals.

Prior to playing, there was a familiarization period. During this period, participants first listened to a recording of each piano composition and then practiced playing along. While listening, participants were instructed to remain as still as possible. While practicing, participants were asked to play along with the recorded piano composition and find the beat, or pulse, of the music that felt most natural to them. If participants were not able to identify what a “beat” or “pulse” was in the music, they were given several examples of where the beat can fall in music. Participants then continued practicing until they identified a beat. For example, during a piano composition with four beats per measure, a participant may choose to play all four downbeats or only the first and third beats in each measure, among other options. Practicing continued until participants identified a steady beat that they preferred to play, which synchronized with the pulse of the music, and which they felt able to maintain for the duration of the piano composition.

After the familiarization period, participants were instructed to play the drum along with the piano composition by following one of two *music playing conditions*: (1) Beat or (2) Improvise. During the *Beat* condition, participants were instructed to play a rhythm that synchronized with the pulse of the music and which they preferred to play. Participants were not required to play the same beat as they identified in the familiarization period, and they were not required to choose the same beat each time they played. However, each time they selected a beat to play during the Beat condition, participants were asked not to change the rhythm they played as much as possible for the duration of the piano composition. In contrast, during the *Improvise* condition, participants were invited to vary their rhythm freely, as desired for personal expression while playing with the piano compositions. In this way, the Beat and Improvise conditions both represented personal preference, but only the Beat condition was constrained to stay the same for the duration of each piano composition. Participants played the drum with the piano compositions with one of two *accompaniment conditions* (1) Recorded or (2) Live. During the *Recorded* condition, accompaniment was provided from a recording of the piano compositions. The same recordings were used for all participants (see Audio S1, S2, S3, and S4 in Supplementary Information for recordings). During the *Live* condition, piano compositions were performed live by the same music therapist who played in the recordings. During the Beat condition, the live accompaniment was played following the piano composition as closely as possible. During the Improvise condition, the music therapist played the piano composition responsively, to reflect the improvised rhythmic patterns played by the participant. In allowing responsive accompaniment during the Improvise condition, we aimed to provide findings that meaningfully inform the implementation of music interventions in real world settings. When music therapists play live accompaniment with improvisation, the intention is to improvise collaboratively with clients, supporting a process of music co-creation^59^. As such, responsive playing during the Improvise condition followed guidelines used in clinical music therapy to support participant’s sense of being heard and supported in their music making^62^. For example, in some cases, the music therapist slightly altered the rhythmic phrasing over several measures to imitate a pattern played by a participant. In this way, the Beat and Improvise conditions applied to both the participant and the accompaniment, where improvisational freedom was shared between the two musical parts. However, to allow comparison between conditions, we constrained the deviance in the live accompaniment from the original piano composition during the Improvise condition. Responsive playing was required to stay within the overall structure of the composition, such that the melodic, harmonic, and rhythmic phrasing retained the overall contour of the original composition (see Audio S5, S6, S7, and S8 in Supplementary Information for a representative example of a participant playing all four conditions, including Recorded and Live accompaniment conditions; see Audio S9, S10, S11, and S12 in Supplementary Information for a second representative example of a participant playing all four conditions). The experimental design aims to strike a balance that allows for meaningful comparison to real world uses of music in clinical interventions while also maintaining comparable musical conditions.

The music therapist remained in the room in all conditions, regardless of whether a recording was being played. Piano and drums were played on Musical Instrument Digital Interface (MIDI) devices, including the Roland SPD-One Percussion MIDI drum pad, tuned to the sound of snare drum, and a Roland FP-60X  digital keyboard. Both MIDI inputs were recorded to individual tracks in Logic Pro, with real time sound output to a Roland KC-400 150 W 12-inch keyboard amp.

### Measures

Baseline measures evaluated demographic characteristics of participants and musical reward-sensitivity and training. During the music playing task, motor (EMG, accelerometry), and autonomic (EDA, ECG) data were collected using a MP160 data acquisition module (BIOPAC Systems Inc.) and AcqKnowledge software (see additional details below). The drum strike count was calculated using MIDI recordings. All signal processing for this data was completed in MATLAB R2022b. Custom code for processing of physiological data in MATLAB is available upon request to the first author.

### Baseline characteristics

Baseline screening included demographic information—including age, gender, race, ethnicity, and education level. Baseline screening also measured factors known to play a role in response to music-playing tasks, including sensitivity to musical reward and musical training. Sensitivity to musical reward was measured via the Barcelona Music Reward Questionnaire (BMRQ), a reliable five-factor scale^11^. Music training was measured via the music training subscale of the Goldsmith Music Sophistication Index (Gold-MSI), a self-report scale with strong internal consistency (Cronbach’s alpha = 0.903)^63^. We also used the Gold-MSI to assess music genre preference over three categories: Rock/Pop, Classical, and Jazz.

### Behavioral correlates of the music playing experience

After each music-playing task, participants provided responses for twelve questions using a 5-point Likert scale. These questions were related to their affective and physical response, as well as their perception of the music (Table 1). Questions were drawn from variables previously related to brain activity in reward- and emotion-related brain regions during music tasks^1,4,7,64^, behaviorally associated with reward sensitivity^65^, and related to moving to music and groove^14,66^. We included difficulty and fatigue as controls for questions assessing positive associations with moving to music. We also queried creative processes which may relate to music improvisation^31,34^. Principal component analysis (PCA) was used to identify the main components of participant responses across these questions. The number of main components were determined using parallel analysis. Participant loadings on these main components, rather than responses to each Likert scale question, were analyzed in relation to participant experience of conditions (see Results).

**Table 1:** Experience of music survey items.

How much pleasure did you experience?
How much difficulty did you experience?
How much fatigue did you experience?
How much did you feel in synchrony with the music?
How familiar was the music?
How motivated did you feel to play?
How surprised were you by the music?
How activated did you feel?
How absorbed did you become?
How creative did you feel?
How much momentum did the music provide for you?
How active were you?

Using a Likert Scale of 1 (“none/not at all”) to 5 (“a lot/very much”) participants were asked to rate their experience after each music playing task.

### Success of music playing

To confirm the success of the music playing task across conditions, we assessed individual ratings of synchrony as a separate analysis from the PCA. Recent research has shown that perceived synchrony (measured through subjective behavioral ratings) during music playing correlates more strongly with groove than measured synchrony from the music itself^67^. Here we use perceived synchrony as a measure of the success of the music playing tasks. We used linear mixed models to predict behavioral synchrony ratings in a model with the music playing condition (Improvise vs. Beat), the music accompaniment condition (Live vs. Recorded), and their interaction as fixed effects, with musical training, sensitivity to music reward, and piano composition style as control variables, and a random intercept for subject^68^. Results are presented in Supplementary Information and show that the Improvise condition during Recorded accompaniment had the lowest ratings. However, perceived synchrony levels in this condition remained close to a neutral perception of synchrony (where a rating of 3 indicates neither disagreeing nor agreeing with the perception of synchrony), on average. This demonstrates that participants generally did not feel out of synchrony and suggests the overall success of music playing (see Supplementary Fig. S2).

### Electromyography

EMG was measured via surface electrodes placed over the wrist flexor carpi radialis and extensor carpi radialis longus on the arm used to play the drum. Prior to electrode placement, the skin was cleaned with alcohol. EMG signals were filtered with a 10–400 Hz band-pass filter, constructed by convolving a 2nd order high pass Butterworth filter and 3rd order low pass Butterworth filter^69^. The signals were then rectified by taking the root mean square of 10 ms windows of the signal. Signals were normalized by dividing by the participant’s maximum voluntary contraction (MVC) ^69^. The MVC was measured by providing resistance to movement at about 45° of rotation through wrist extension and flexion, during which participants were asked to exert the maximum possible force against the resistance. MVC signals were filtered and rectified as described above, and the final MVC value was calculated as the average amplitude of the maximum one-second region of the signal.

To ensure background noise did not influence the overall EMG average, movement-related peaks in normalized EMG amplitude were identified by alignment with peaks in the Z component of the accelerometry signal (see below) using custom code. The lowest 10% of peaks in EMG amplitude were dropped to eliminate noise, and plots were visually inspected to ensure proper alignment between EMG and accelerometry signals. The average of movement-related peaks in EMG amplitude was calculated. Since participants played the drum with slightly different arm positions, the results present findings for the average wrist flexor and extensor activation, rather than considering each muscle separately. EMG signals were recorded at a sampling rate of 2000 samples/second.

### Accelerometry

A triaxial accelerometer was secured to the back of the hand playing the drum using a Velcro strap, with placement under the metacarpophalangeal joints and between the third and fourth fingers. Accelerometry signals were acquired at a sampling rate of 2000 samples/second, except for the first 14 participants, for whom the y component was sampled at 250 samples/second due to a technical problem. To correct for this, all other accelerometry signals were down sampled to 250 Hz. A 4th-order Butterworth high pass filter with a cut-off at 0.5 Hz was then used to remove the gravitational component^70^. Leading and trailing samples were dropped due to filter and resampling artifacts, with an overall sample loss of less than 10% of the signal. The x, y, and z components of the resulting accelerometer signal were then transformed into magnitude of acceleration taking the square root of the sum of the squares of each component. The total acceleration was calculated as the sum of the magnitude of acceleration over the entire signal^70^.

### Drum strike count

The drum strike count (i.e., the total number of taps on the drum) during each music condition was calculated from the MIDI recordings. MIDI files were loaded into MATLAB, and the MIDI toolbox 1.1 was used to calculate the drum strike count^71^.

#### Association between motor outcomes

Total acceleration was significantly associated with mean muscle activation and the drum strike count, but there was not a significant association between mean muscle activation and the drum strike count (see Supplementary Information). However, we chose to maintain all three measures as we think they provide different characteristics of motor activity. Total acceleration measures the cumulative activity over the entire one-minute task for each condition. By comparison, mean muscle activation is averaged over each movement, and therefore provides a measure that is independent of variability in how many times participants played the drum in each condition. Finally, whereas total acceleration measured continuous movement in 3 dimensions, the drum strike count provided discrete counts of how many times participants played the drum.

#### Electrodermal activity

EDA electrodes were placed on the index and middle finger of the hand not playing the drum, with the hand resting on a table. We instructed participants to move the hand with the EDA electrodes as little as possible to avoid motion artifacts. Tonic response of EDA was calculated as the mean of the signal for each one-minute trial^72^. Music-playing responses were normalized for each participant by subtracting the tonic response to the listen-only condition from each trial^72^. Six participants were dropped from the EDA analysis due to hyperhidrosis that caused ceiling effects in the EDA signal (n = 5) or insufficient signal (n = 1).

#### Electrocardiography

ECG was recorded using three electrodes placed on the torso. Raw ECG signals were detrended, and heartbeats were identified as the R wave maxima of the QRS complexes. First, the signal was detrended and local maxima were identified using built-in Matlab functions. Then, to remove duplicate local maxima clustered at R wave maxima, we used custom code to create one second windows around each local maxima, and then removed maxima below a threshold of 80% of the highest point in the 1 s window. Finally, ECG signals were visually inspected to ensure that QRS complexes were accurately identified. Three participants were dropped from ECG analyses due to insufficient signal-to-noise ratio. Beats per minute (BPM) were calculated as the sampling rate (in samples/sec) divided by the average samples per beat, multiplied by 60 (sec/min)^73^. Heart rate variability (HRV) was calculated as the root mean square of successive differences (RMSSD) between R wave maxima^74,75^. ECG signals were recorded at 2000 samples/second.

#### Experimental design and statistical analysis

**Figure 1:**
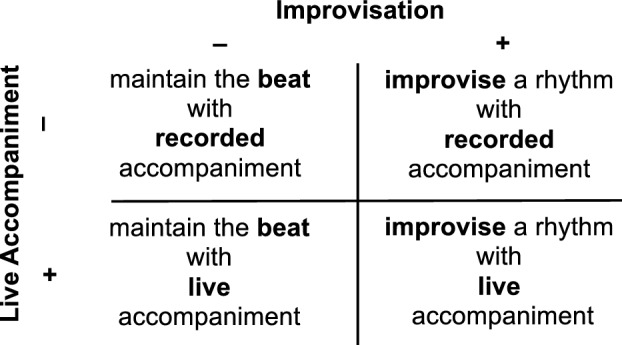
Experimental Design. Using a 2 × 2 crossover design, each participant completed all four combinations of the music playing tasks, including with or without improvisation (music playing conditions: Improvise vs. Beat) and with or without live accompaniment (accompaniment conditions: Live vs. Recorded).

Using a crossover design, all participants completed all four combinations of music playing and accompaniment conditions (Fig. 1) with each of the four piano compositions, for a total of 16 music playing trials. Participants were randomized to one of four condition orders (Figure S1 in Supplementary Information). The same condition order was repeated for each of the four piano compositions, and all conditions were completed for one composition before moving to the next. Outcomes included behavioral responses (first and second principal components of questionnaire responses), motor responses (EMG, accelerometry, and drum strike count), and autonomic responses (tonic response of EDA, BPM, and HRV). For each outcome, linear mixed models were calculated with fixed effects including the music playing condition (Improvise vs. Beat), the music accompaniment condition (Live vs. Recorded), and their interaction, with control variables for musical training, sensitivity to music reward, and piano composition style, and a random intercept for subject^68^. For EDA, BPM, and HRV, total acceleration was additionally included in the model as a control, to eliminate motion as an explanation for increased autonomic response. Post-hoc comparisons of significant effects were adjusted for multiple tests using the Tukey method^76^.

To evaluate the association between reward-related behavioral measures and motor responses (accelerometry, EMG, and drum strike count), we again used linear mixed models^68^. As a measure of reward, we used the first principal component of participant responses to the 12-item survey related to the music playing experience. This principal component includes positive loadings for pleasure, motivation, activation, and momentum–responses related to the hedonic (i.e., liking) and motivation (i.e., wanting) components of reward processing^53^. This first component therefore provides a broader composite measure of reward than the single behavioral rating of pleasure, as it encompasses two of the main components of reward (i.e., liking and wanting) experienced by the participants Fixed predictors included motor response (we computed 3 different models for our 3 different measures: accelerometry, EMG, and drum strike count), music playing condition (Improvise vs. Beat), music accompaniment condition (Live vs. Recorded), and their three-way interaction, as well as control variables for musical training, sensitivity to music reward, and piano composition style, with a random intercept for subject. The three-way interaction in this model accounts for potential modulation of the association between motor responses and reward by the four music tasks. Post-hoc comparisons of linear trends were evaluated by averaging over other factor level predictors in the model^76^. Bonferroni correction was used to account for multiple comparisons. There was a consistent pattern of results across motor responses before Bonferroni correction (see Results section). Since Type I error is minimized when all responses show a similar effect, we have elected to report significance levels with and without Bonferroni correction to provide a more complete summary of these results, which we believe are relevant to inform future research.

For all analyses of linear mixed models, Wald Chi Square tests were used to evaluate significance of main effects and interactions. Plots include error bars based on 95% confidence intervals. All analyses were done in R (packages: psych, factoextra, lme4, foreign, aod, car, emmeans, emtrends, ggplot2, ggeffects).

## Results

A summary of main results can be found in Table 2.

**Table 2:** Predictor significance for behavioral, motor, and autonomic response to music playing.

	Predictors						
	Playing Condition (Improvise) χ^2^(p)	Accompaniment (Live) χ^2^(p)	Improvise x Live χ^2^(p)	Composition Style χ^2^(p)	BMRQ χ^2^(p)	Gold-MSI χ^2^(p)	Total Acceleration χ^2^(p)
Behavior							
Reward	**15.18 (< 0.001)**	**7.73 (0.005)**	**14.53 (< 0.001)**	**33.92 (< 0.001)**	**4.51 (0.034)**	0.20 (0.659)	NA
Challenge	**50.12 (< 0.001)**	0.90 (0.344)	0.01 (0.916)	**35.42 (< 0.001)**	1.16 (0.281)	0.64 (0.424)	NA
Motor Response							
Total Acceleration	0.77 (0.382)	0.001 (0.982)	**7.54 (0.006)**	**23.99 (< 0.001)**	1.16 (0.282)	0.73 (0.394)	NA
Mean EMG	**18.11 (< 0.001)**	0.13 (0.720)	**6.12 (0.013)**	**82.60 (< 0.001)**	0.05 (0.816)	0.30 (0.581)	NA
Drum Strike Count	**23.63 (< 0.001)**	0.20 (0.652)	1.43 (0.232)	**61.13 (< 0.001)**	3.64 (0.056)	0.01 (0.909)	NA
Autonomic Response							
Tonic EDA	**5.74 (0.017)**	0.05 (0.820)	0.63 (0.427)	**8.86 (0.031)**	0.005 (0.944)	0.45 (0.504)	**6.22 (0.013)**
BPM	**21.34 (< 0.001)**	1.39 (0.238)	0.15 (0.701)	7.48 (0.058)	0.416 (0.519)	**4.35 (0.037)**	**13.45 (< 0.001**)
HRV	**4.66 (0.031)**	0.20 (0.655)	0.66 (0.418)	3.92 (0.271)	0.002 (0.961)	3.67 (0.055)	**10.16 (0.001)**

Significant effects marked in **bold**.

Interaction effect marked with an “x.”

*BMRQ *Barcelona Music Reward Questionnaire, *Gold-MSI* Goldsmiths Musical Sophistication Index, *EMG* electromyography, *EDA* electrodermal activity, *BPM* beats per minute, *HRV* heart rate variability.

### Participant musical background

Participant scores on the BMRQ (M = 84.93, SD = 9.08) were in the range of the general population (from the original BMRQ paper: M = 78.42, SD = 10.47, n = 857)^11^. Similarly, participant scores on the Gold-MSI musical training subscale (M = 25.40, SD = 10.82) were in the range of the general population (from the original Gold-MSI paper: M = 26.52, SD = 11.44, n = 147,633)^63^. These findings indicate our sample approximates the general population in relationship to sensitivity to musical reward and musical training. Most participants reported music genre preference as Rock/Pop (77%) followed by Jazz (13%) and Classical (10%).

### Behavioral correlates of the music playing experience

**Figure 2:**
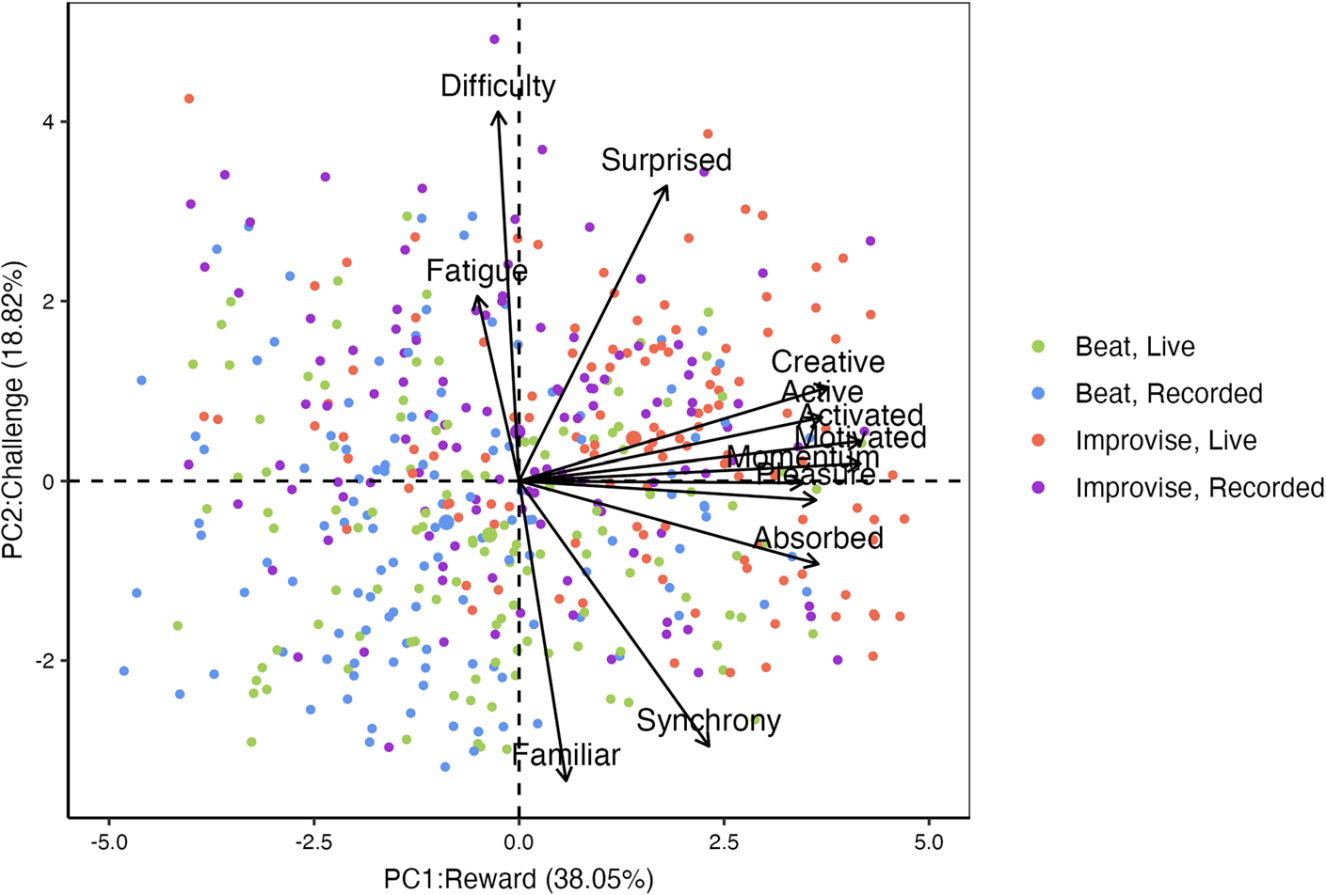
Biplot of principal components derived from the 12-item music experience survey. The first principal component includes responses related to reward, accounting for 38.05% of the variance in survey responses. The second principal component includes responses related to challenge and accounted for 18.82% of the variance in participant responses.

PCA of responses revealed two main components of music playing experience (Fig. 2). The first component (PC1) accounted for 38.05% of the variance in the survey responses and was termed “reward” to summarize positive loadings for participant experiences of feeling pleasure, momentum, activated, motivated, creative, active, and absorbed. The second component (PC2) accounted for 18.82% of the variance in the survey responses and was termed “challenge” to summarize positive loadings of participant experiences of difficulty, fatigue, and surprise, and negative loadings of participant perception of the music as familiar and in synchrony with their playing.

**Figure 3:**
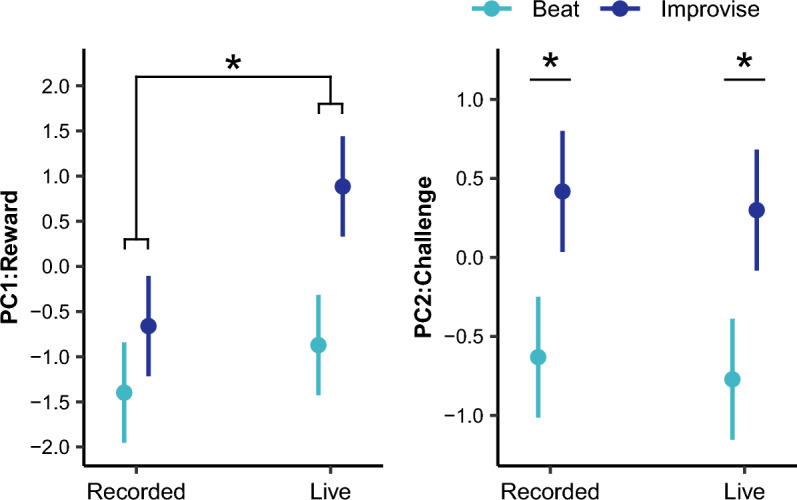
There was a significant interaction between music playing condition and music accompaniment condition on reported reward, such that the combination of Improvise and Live conditions produced the greatest reported reward compared to the other three condition combinations. There was a main effect of music playing condition on reported challenge, such that challenge was greater during the Improvise as compared to the Beat condition. *P < 0.05 after correction for multiple tests.

For reported reward (see Fig. 3 and Table 2), there were significant main effects of music playing condition (Improvise vs. Beat) and accompaniment condition (Live vs. Recorded), as well as a significant interaction between playing condition and accompaniment condition. There were also significant main effects of piano composition style and sensitivity to musical reward (BMRQ), but no significant effect of musical training (Gold-MSI). Pairwise comparisons for music playing conditions, averaged over the levels of piano composition style and corrected for multiple tests, revealed increased reported reward for the Improvise as compared to the Beat condition, for both Live (t(444) = 9.29, *P* < 0.00001) and Recorded (t(444) = 3.90, *P* = 0.0007) accompaniment conditions. Additionally, reported reward increased for the Live as compared to the Recorded condition, for both Improvise (t(444) = 8.17, *P* < 0.00001) and Beat (t(444) = 2.78, *P* = 0.03) music playing conditions. Finally, the combination of Improvise with Live conditions produced the highest levels of reported reward compared to the other condition combinations, including Improvise with Recorded (t(444) = 8.17, *P* < 0.00001), Beat with Live (t(444) = 9.29, *P* < 0.00001), and Beat with Recorded (t(444) = 12.07, *P* < 0.00001) condition combinations. For pairwise comparisons of piano composition style, see Supplementary Information.

With reported challenge as the outcome (see Fig. 3 and Table 2), there was a significant main effect for music playing condition, but not for accompaniment condition, or for the interaction between music playing and accompaniment conditions. There was also a significant main effect of piano composition style, but no significant effect of music training or sensitivity to musical reward. Pairwise comparisons, averaged over the levels of piano composition style and corrected for multiple tests, revealed higher levels of reported challenge for the Improvise as compared to Beat condition, for both Live (t(444) = 7.23, *P* < 0.00001) and Recorded (t(444) = 7.08, *P* < 0.00001) accompaniment conditions. For pairwise comparisons of piano composition style, see Supplementary Information.

### Motor response to music conditions

**Figure 4:**
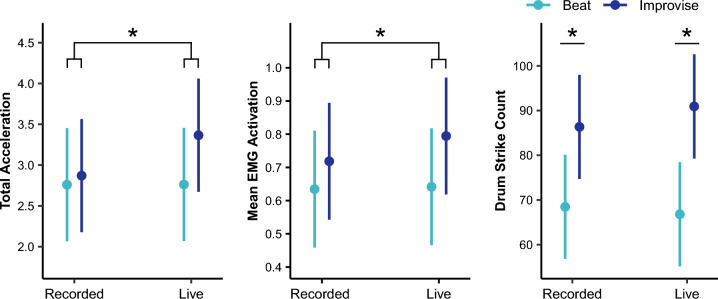
There was a significant interaction between music playing condition and accompaniment condition on total acceleration and mean EMG activation, such that the combination of the Improvise and Live conditions produced the greatest motor response. There was a main effect of music playing condition on drum strike count, such that a greater number of drum strikes were produced during the Improvise as compared to Beat condition. *P < 0.05 after correction for multiple tests.

For total acceleration (see Fig. 4 and Table 2), although there were no main effects for music playing condition or accompaniment condition, there was a significant interaction between music playing condition and accompaniment condition. There was also a significant main effect for piano composition style, but no significant main effect for music training or sensitivity to musical reward. Pairwise comparisons, averaged over the levels of piano composition style and controlling for multiple tests, revealed that the combination of the Improvise with Live conditions produced greater total acceleration than the other three condition combinations, including Improvise with Recorded (t(432) = 3.90, *P* = 0.006), Beat with Live (t(432) = 4.76, *P* = 0.00002), and Beat with Recorded (t(432) = 4.78, *P* = 0.00001) condition combinations. For pairwise comparisons of piano composition style, see Supplementary Information.

For mean EMG activation (see Fig. 4 and Table 2), there was a significant main effect of music playing condition, but not of accompaniment condition. There was a significant interaction between music playing condition and accompaniment condition. There was also a significant main effect of piano composition style, but no significant effects of musical training or sensitivity to musical reward. Pairwise comparisons, averaged over the levels of piano composition style and controlling for multiple comparisons, showed greater mean EMG activation for the Improvise as compared to Beat conditions for both Live (t(372) = 7.76, *P* < 0.00001) and Recorded (t(372) = 4.26, *P* = 0.0002) accompaniment conditions. In addition, the combination of the Improvise with Live conditions produced greater mean EMG activation compared to the other three condition combinations, including Improvise with Recorded (t(372) = 3.86, *P* = 0.0008), Beat with Live (t(372) = 7.76, *P* < 0.00001), and Beat with Recorded (t(372) = 8.11, *P* < 0.00001) condition combinations. For pairwise comparisons of piano composition style, see Supplementary Information.

For drum strike count (see Fig. 4 and Table 2), there was a main effect of music playing condition, but not of accompaniment condition. There was no significant interaction between music playing and accompaniment conditions. There was also a significant main effect of piano composition style and a trend towards a main effect of sensitivity to musical reward. There was no significant effect of musical training. Pairwise comparisons in music playing condition, averaged over the level of piano composition style and controlling for multiple tests, revealed a greater number of drum strikes played during the Improvise as compared to Beat conditions, for both the Live (t(444) = 4.86, *P* < 0.00001) and Recorded (t(444) = 6.55, *P* < 0.00001) accompaniment conditions. For pairwise comparisons of piano composition style, see Supplementary Information.

### Autonomic response to music conditions

**Figure 5:**
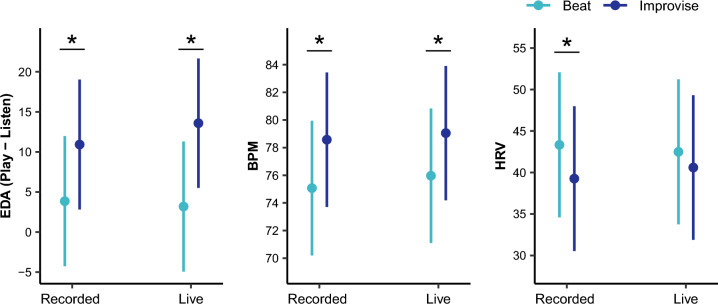
There was a main effect of music playing condition on autonomic response, such that the Improvise condition led to increased autonomic arousal as measured by increased tonic EDA and BPM, and parasympathetic withdrawal as measured by decreased HRV, as compared to the Beat condition. Pairwise comparisons revealed higher EDA and BPM for the Improvise as compared to Beat condition during both Recorded and Live accompaniment conditions. For HRV, the difference between the Improvise and Beat conditions was only significant in the Recorded condition. *P < 0.05 after correction for multiple tests.

For tonic EDA (see Fig. 5 and Table 2), there was a significant main effect of music playing condition, but not of accompaniment condition. Note that we controlled for the effects of movement by including total acceleration in the model. There was no significant interaction between music playing and accompaniment conditions. There were also significant main effects of total acceleration and piano composition style, but no significant effects of musical training or sensitivity to musical reward. Pairwise comparisons for music playing conditions, averaged over the levels of piano composition style and controlling for multiple testing, revealed greater tonic EDA, indicating higher autonomic arousal, for the Improvise as compared Beat condition for both Live (t(345.94) = 3.47, *P* = 0.0006) and Recorded (t(341.19) = 2.40, *P* = 0.017) accompaniment conditions. For pairwise comparisons of piano composition style, see Supplementary Information.

For BPM (see Fig. 5 and Table 2), there was a main effect of music playing condition, but not of accompaniment condition. Here, we also controlled for the effects of movement by including total acceleration in the model. There was no significant interaction between music playing condition and accompaniment condition. There were also significant main effects of total acceleration and musical training, but no significant effects of piano composition style or sensitivity to musical reward. Pairwise comparisons for music playing condition, averaged over the level of piano composition style and controlling for multiple testing, revealed greater BPM, indicating higher autonomic arousal, for the Improvise as compared to Beat conditions for both Live (t(398.65) = 3.95, *P* = 0.00009) and Recorded (t(397.07) = 4.62, *P* = 0.00001) accompaniment conditions.

For HRV (see Fig. 5 and Table 2), there was a significant main effect of music playing condition, but not of accompaniment condition. We also controlled for the effects of movement by including total acceleration in the model. There was no significant interaction between music playing and accompaniment conditions. There was also a significant main effect of total acceleration, but no significant effects of piano composition style, musical training, or sensitivity to musical reward. Pairwise comparisons for music playing condition, averaged over the levels of piano composition style and controlling for multiple testing, revealed lower HRV, indicating parasympathetic withdrawal^77^, for the Improvise compared to Beat conditions for the Recorded accompaniment condition (t(397.14) = 2.16*,* *P* = 0.031). Although HRV was also lower for the Improvise compared to Beat conditions for the Live accompaniment condition, this difference was not significant (t(400.12) = 0.976*,* *P* = 0.330).

### Association between reward and motor responses

The association between reported reward (i.e., the first component of the PCA) and each motor response (total acceleration, mean EMG activation, and drum strike count) was evaluated by adding the motor response to the linear mixed model predicting reported reward. The main effect of motor response was considered, as well as the three-way interaction between motor response, music playing condition, and accompaniment condition, the main effect for control variables for piano composition style, musical training, and sensitivity to musical reward, and a random intercept for subject. To account for multiple comparisons, significance levels with and without Bonferroni correction are reported for the association between reported reward and the three motor responses.

**Figure 6:**
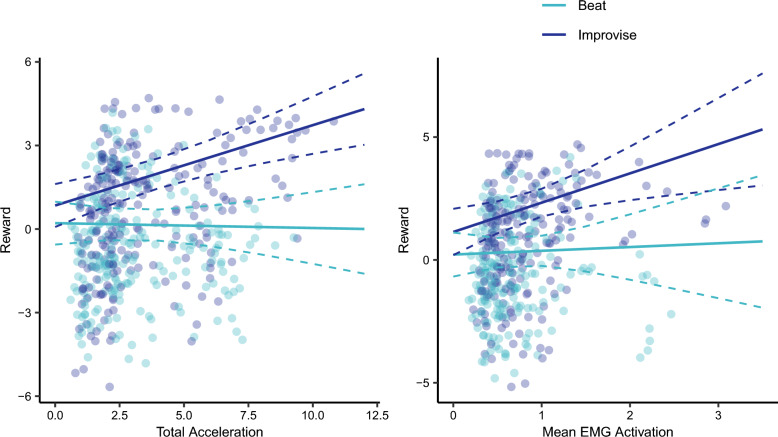
There was a significant interaction between total acceleration and music playing condition on reported reward, such that reward was positively associated with total acceleration for the Improvise condition but not the Beat condition. This interaction effect remained significant after Bonferroni correction for multiple comparisons. There was also a significant interaction between mean EMG activation and music playing condition on reported reward, such that reward was positively associated with mean EMG activation for the Improvise condition but not the Beat condition. This interaction effect approached significance after Bonferroni correction for multiple comparisons.

There was no significant main effect of total acceleration on reported reward (χ^2^ (1) = 0.04, *P* = 0.841) and no significant triple interaction between total acceleration, music playing condition, and accompaniment condition on reported reward (χ^2^ (1) = 0.0004, *P* = 0.983). However, there was a significant interaction effect between total acceleration and music playing condition on reported reward (Fig. 6; χ^2^ (1) = 12.15, *P* = 0.0005). This effect remained significant after Bonferroni correction for multiple comparisons (*P* = 0.0015). There was also a significant main effect of piano composition style (see Supplementary Information) and sensitivity to musical reward (χ^2^(1) = 3.92, *P* = 0.048). Notably, previously significant effects became non-significant after including total acceleration in the model predicting reported reward, including the interaction between music playing condition and accompaniment condition (χ^2^ (1) = 3.05, *P* = 0.081) and the main effects of music playing condition (χ^2^ (1) = 3.34, *P* = 0.841) and accompaniment condition (χ^2^ (1) = 3.46, *P* = 0.063). There was also no significant interaction effect for total acceleration and accompaniment condition (χ^2^ (1) = 0.098, *P* = 0.755) and no main effects of musical training (χ^2^ (1) = 0.533, *P* = 0.465). Simple effects revealed a positive relationship between total acceleration and reported reward during the Improvise condition, using the 95% confidence interval to indicate significance (estimated slope = 0.302; 95% CI = 0.166, 0.438), but not for the Beat condition (estimated slope = − 0.003; 95% CI = − 0.153, 0.148). The slopes describing the relationship between total acceleration and reported reward were significantly different between the Improvise and Beat conditions (estimate = 0.305, t(453) = 4.60, *P* < 0.0001).

There was no significant main effect of mean EMG activation on reported reward (χ^2^ (1) = 0.10, *P* = 0.753) and no significant triple interaction effect between mean EMG activation, music playing condition, and accompaniment condition on reported reward (χ^2^ (1) = 0.30, *P* = 0.586). However, there was a significant interaction effect between mean EMG activation and music playing condition on reported reward (Fig. 6; χ^2^(1) = 5.55, *P* = 0.0184), while the interaction between music playing condition and accompaniment condition approached significance (χ^2^ (1) = 3.78, *P* = 0.052). After Bonferroni correction, the interaction between mean EMG activation and music playing condition on reported reward approached significance (*P*  = 0.0552). There was also a significant main effect of music playing condition (χ^2^ (1) = 5.54, *P* = 0.019), piano composition style (see Supplementary Information), and sensitivity to musical reward (χ^2^(1) = 8.89, *P* = 0.003). There was no significant interaction effect for mean EMG activation and accompaniment condition (χ^2^ (1) = 0.07, *P* = 0.786) and no main effects of musical training (χ^2^ (1) = 0.63, *P* = 0.426) or accompaniment condition (χ^2^ (1) = 2.40, *P* = 0.122). Simple effects revealed a positive relationship between mean EMG activation and reported reward during the Improvise condition, using the 95% confidence interval to indicate significance (estimated slope = 1.300; 95% CI = 0.483, 2.117) but not the Beat condition (estimated slope = 0.091; 95% CI = − 0.784, 0.967). The slopes describing the relationship between reported reward and mean EMG activation were significantly different between Improvise and Beat conditions (estimate = 1.21, t(391) = 3.81, *P* = 0.0002).

There was no significant main effect of drum strike count on reported reward (χ^2^ (1) = 0.84, *P* = 0.360) and no significant triple interaction effect between drum strike count, music playing condition, and accompaniment condition on reported reward (χ^2^(1) = 0.0065, *P* = 0.936). The interaction effect between drum strike count and music playing condition on reported reward approached significance (χ^2^ (1) = 3.83, *P* = 0.0503), but was not significant after Bonferroni correction (*P* = 0.151). There was also a significant main effect of piano composition style (see Supplementary Information), and a trend towards a main effect for sensitivity to musical reward (χ^2^(1) = 3.05, *P* = 0.081). Notably, previously significant effects became non-significant after adding drum strike count to the model, including the main effect of music playing condition (χ^2^ (1) = 2.20, *P* = 0.138). There was also no significant interaction effect for drum strike count and accompaniment condition (χ^2^ (1) = 0.204, *P* = 0.651), no significant interaction between music playing condition and accompaniment condition (χ^2^ (1) = 2.04, *P* = 0.154), and no main effects of musical training (χ^2^ (1) = 0.247, *P* = 0.619) or accompaniment condition (χ^2^ (1) = 2.56, *P* = 0.109). Simple effects for the association between drum strike count and reported reward revealed a positive relationship between drum strike count and reported reward during the Improvise condition, using the 95% confidence interval to indicate significance (estimated slope = 0.0133; 95% CI = 0.0078, 0.0189), but not the Beat condition (estimated slope = 0.0046; 95% CI = − 0.0012, 0.0104). The slopes describing the relationship between reported reward and drum strike count were significantly different between the Improvise and Beat conditions (estimate = 0.0087, t(496) = 2.53, *P* = 0.0118).

## Discussion

Here we show that improvisation and live accompaniment increase reported reward and motor performance during a music playing task. The combination of improvisation and live accompaniment led to increased acceleration of hand movements while playing the drum, coupled with increased amplitude of muscle contractions in wrist flexor and extensor muscles. Importantly, increased movement was associated with increased reported reward only when improvising. These findings suggest a mechanism by which combined improvisation and live accompaniment increase reported reward and motivate increased motor activity. We also show that improvisation leads to increased autonomic arousal, as measured by increased tonic EDA and BPM and decreased HRV. Together, these findings have implications for enhancing reward, engagement, and motor performance during music playing tasks that support exercise and physical rehabilitation.

In our experimental design, we did not provide any instructions to participants regarding how much they should move while playing music in any condition. The increased acceleration and muscle activation during conditions with combined improvisation and live accompaniment might thus reflect participants’ intrinsic motivation to move. Supporting this mechanism, participants’ self-reports of pleasure, motivation, momentum, and activation loaded onto a principal component that we termed “reward” which increased for conditions with improvisation and live accompaniment as compared to the other conditions. Further, the association between reported reward and motor response was moderated by the music playing condition, such that increased reward was associated with increased total acceleration of hand movements while improvising, but not while maintaining the beat. The same moderation effect approached significance for mean EMG activation, with simple effects demonstrating that increased mean EMG activation was significantly associated with increased reported reward while improvising, but not while maintaining the beat. This relationship between reward and movement is supported by previous research demonstrating that reward enhances motor performance and learning^28^ and contributes to motor recovery post-stroke^35^. An association between enhanced music reward and improved motor learning has also been identified in a music playing task involving playing a learned melody^78^. In our task, the flexibility of choice provided during improvisation may have played a key role in enhancing reward and movement. Autonomy of choosing what to do has been shown to enhance reward, motor performance, and learning^38–42^. However, it is also possible that the limited variation in movement while maintaining the beat inhibited reward-enhanced movement for the Beat condition. Together, our findings demonstrate that improvisation and live accompaniment may be used to facilitate increased reward and movement during music playing, and that the association between reward and movement is greater during improvisation.

The music playing condition also produced a main effect on average muscle activation, drum strike count, and reported reward, with the Improvise condition associated with greater motor response and reward as compared to the Beat condition. This suggests that improvisation, regardless of live or recorded accompaniment, may increase reward and enhance motor performance. Participants chose their own beat, and therefore set their own pace when maintaining the beat; however, they increased their frequency of movement while improvising, as demonstrated by the increase in the drum strike count. This increase in frequency during improvisation may be partially due to the freedom from constraints in movement frequency, which necessarily limited the number of drum strikes that could be played during the Beat condition. However, the EMG analysis addressed this limitation by averaging peak EMG activation for each movement, thus measuring movement intensity independent of movement frequency. This analysis revealed increased muscle activation for the Improvise condition as compared to the Beat condition, supporting the link between improvisation and enhanced motor performance. These findings support previous studies linking choice during tasks to enhanced motor performance^38,41^, and modulation of reward-related brain areas^40^, suggesting a role of agency in motivating movement. Together, these findings suggest an important role of improvisation for enhancing reward and motor performance, with mechanisms that may relate to increased autonomy and agency^38–42^.

The accompaniment condition produced a main effect on reported reward, with live accompaniment producing the greater reward compared to recorded accompaniment. However, there were no main effects of the accompaniment condition on motor or autonomic responses. This suggests that the benefits of live accompaniment are best realized in combination with improvisation, as the interaction between live accompaniment and improvisation led to the greatest levels of reported reward and enhanced motor performance. Social cooperation has demonstrated benefits for improving intrinsic motivation and motor learning^48^, and the present findings suggest that the combined effects of live accompaniment and improvisation supported a cooperative effort to coordinate drum and piano playing. Importantly, the piano accompaniment for this study was provided by a music therapist with specialized training in clinical improvisation, which emphasizes responsiveness to participant playing to facilitate a supportive, emotionally attuned music playing experience^62^. Together, these results suggest that live accompaniment, on its own, does not enhance motor performance, but that live accompaniment can enhance the benefits of improvisation for motor performance through a mechanism of intrinsic reward that might be related to social cooperation.

We also demonstrated that improvisation during music-playing increased autonomic arousal, as measured via tonic EDA, BPM, and HRV, as well as the participant reports related to challenge. Importantly, the association between improvisation and autonomic arousal remained significant after controlling for movement, suggesting that arousal relates to mechanisms beyond physical exertion^79^. Given the association between improvisation and challenge, it may be that the increased autonomic arousal during improvisation relates to cognitive control. Supporting this potential mechanism, rhythmic improvisation has previously been linked to increased activation in the dorsal anterior cingulate cortex (dACC) when compared to repeating a rhythm^45^, and previous research links dACC activation to cognitive control^80^ and evaluation of creative tasks^81^. However, music improvisation has also been linked to activation in the default mode network and deactivation of the executive network, with the extent of executive network deactivation shown to vary with the level of expertise in music improvisation^44,82^. These findings highlight the complexity of improvisation as a creative task that involves both idea generation and evaluation^37,81^. Our findings suggest that cognitive challenge played a role in rhythmic improvisation for our participants, an effect that remains significant while controlling for the level of musical expertise.

Finally, our analyses demonstrate that the piano composition style significantly modulated participant reports related to reward and challenge, motor response, and autonomic response during music playing. Notably, reward ratings and mean EMG activation were lower for the March compared to Rock and Salsa compositions. Similarly, reward ratings and mean EMG activation were lower for the Waltz compared to the Rock composition. There was a trend toward this pattern of response for the Waltz and Salsa. This pattern suggests that piano composition styles with lower reward ratings also produced lower muscle activation during each movement. These effects may relate to familiarity with musical styles and the amount of musical groove^14,64,66,83^. Total acceleration and drum strike count were also lower for the March compared to the other three piano compositions styles, suggesting that lower reward ratings for the March also reduced the total motor activity and the number of drum strikes played. However, this effect was not present for the Waltz, suggesting that other factors such as tempo and the strength of the pulse on each downbeat (i.e., feeling the beat on the 1st downbeat only vs feeling the beat on the 1st and 3rd, for example) may have influenced how often participants played the drum during each piano composition. The effects of piano composition style in this study suggests future research should examine how to manipulate music composition characteristics to enhance motor and affective outcomes during music playing.

Together, our findings suggest two distinct mechanisms influencing response to music-playing tasks. First, reward during music-playing is facilitated by the combination of improvisation and live accompaniment. During improvisation, reward is associated with increased acceleration of hand movements and approaches significant association with muscle activation. Second, improvisation increases challenge and autonomic arousal while controlling for motor output, suggesting increased attentional processes when generating musical ideas. These findings have important implications for increasing reward and attention during music-playing tasks, which may promote engagement, dose of movement, and improved outcomes during exercise and physical rehabilitation. Understanding the link between music reward and movement is essential for improving our general understanding of the intricate relationship between motor and reward-related regions and their neural substrates^84^. Future studies should use neuroimaging methods to investigate functional connectivity between reward and sensorimotor regions during music playing, and to better understand the relationship between improvisation, cognitive challenge, and executive function. Additionally, studies investigating these mechanisms in clinical populations have potential to inform the development of music playing tasks to optimize motor rehabilitation^85^.

### Supplementary Information

Supplementary Information 1.

Supplementary Information 2.

Supplementary Audio 1.

Supplementary Audio 2.

Supplementary Audio 3.

Supplementary Audio 4.

Supplementary Audio 5.

Supplementary Audio 6.

Supplementary Audio 7.

Supplementary Audio 8.

Supplementary Audio 9.

Supplementary Audio 10.

Supplementary Audio 11.

Supplementary Audio 12.

## Data Availability

The dataset analyzed during this study are included in this published article (and its Supplementary Information files).
