# Supplementary material for: Improvisation and live accompaniment increase motor response and reward during a music playing task
Source: Sci Rep. 2024 Jun 7;14:13112. doi: 10.1038/s41598-024-62794-6 (PMC11161496; doi:10.1038/s41598-024-62794-6)
Supplement: Supplementary file 2 — Supplementary Information 2. [file 41598_2024_62794_MOESM2_ESM.pdf]

Improvisation and Live Accompaniment Increase Motor Response and Reward During a Music Playing Task

Anna Palumbo, Karleigh Groves, Eva Luna Munoz-Vidal, Alan Turry, Robert Codio, Preeti Raghavan, Heidi Schambra, Gerald T. Voelbel, Pablo Ripollés

Supplementary Materials

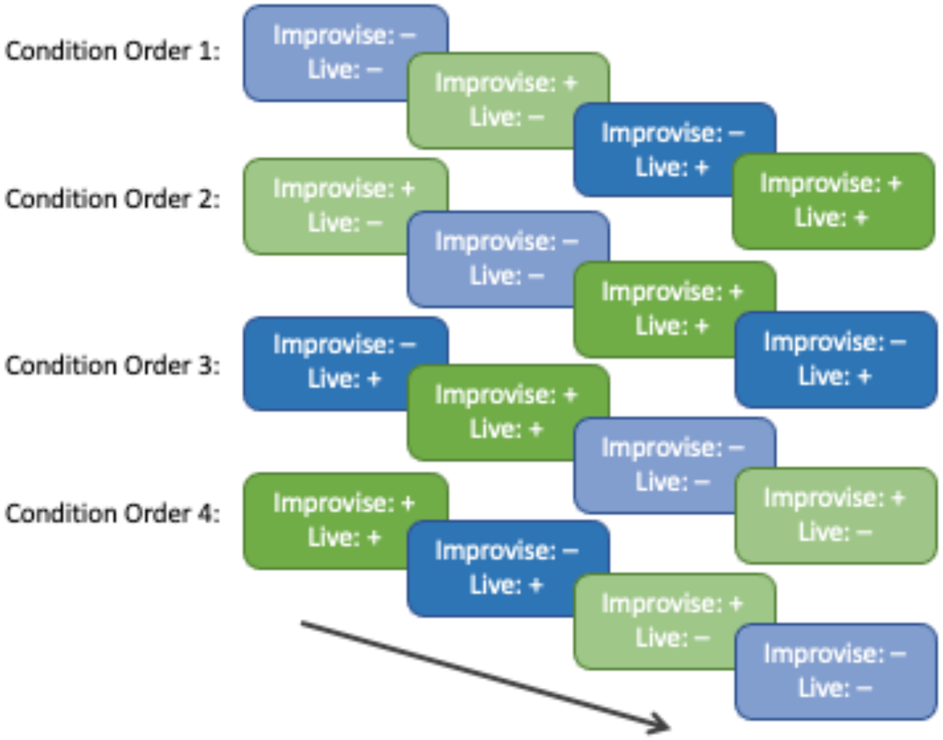

Figure S1: Condition Orders

Participants were randomized to one of four condition orders. Each condition order alternated between music playing conditions (Improvise and Beat), with half of the condition orders starting with improvisation. Accompaniment conditions (Live and Recorded) were completed consecutively, such that participants completed both music playing conditions with one accompaniment condition and then repeated both music playing conditions with the other accompaniment condition. Half of the condition orders started with live accompaniment. The same condition order was repeated for each of the four piano compositions played by each participant.

Supplementary Results

Success of Music Playing Across Conditions as Measured by Perceived Synchrony

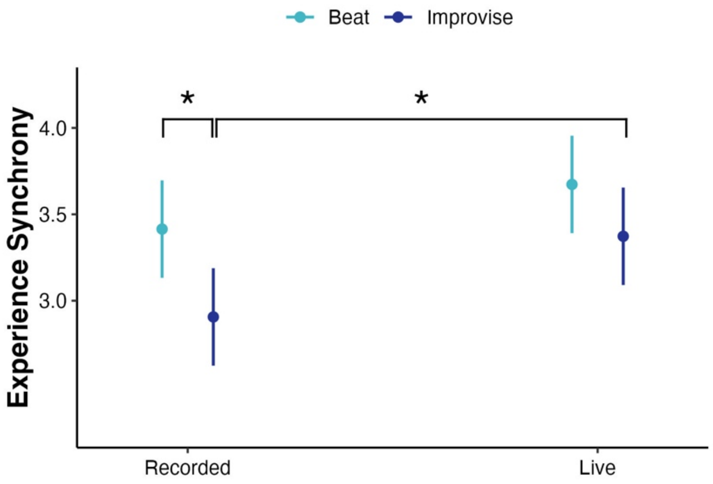

Figure S2: Experience of Synchrony Across Music Playing Conditions
Participants reported the lowest synchrony ratings during the Improvise Condition with Recorded accompaniment. However, synchrony ratings during this condition remained close to a neutral 3 in a 1-5 scale (where a rating of 3 indicated neither disagreeing or agreeing with the perception of synchrony), suggesting participants generally did not feel out of synchrony and that there was an overall perception of success of music playing across conditions

There were significant main effects of music playing condition (Improvise vs. Beat: χ^2 (1) = 17.12, p = 0.00004), accompaniment condition (Live vs. Recorded: χ^2 (1) = 4.42, p = 0.035), piano compositions style (χ^2 (3) = 14.44, p = 0.0024), and sensitivity to musical reward (χ^2 (1) = 6.00, p = 0.014) on synchrony ratings. There was no significant main effect for music training and no significant interaction between music playing condition and accompaniment condition. Pairwise comparisons of music playing condition revealed lower synchrony ratings for the Improvise as compared to the Beat condition during Recorded (t(444) = 4.13, $p < 0.0002$) but not Live (t(444) = 2.44, p = 0.071) accompaniment conditions. Similarly, synchrony ratings were greater during Live as compared to Recorded accompaniment conditions for the Improvise (t(444) = 3.80, p = 0.00095) but not Beat (t(444) = 2.10, p = 0.154) music playing conditions. We interpret these findings as demonstrating participants generally felt confident maintaining the beat during recorded or live accompaniment, and therefore experienced stable levels of synchrony. However, when improvising, participants felt more synchronized when there was mutual adaptation with the accompaniment (i.e., during the Live condition). The lowest synchrony ratings occurred in the Improvise condition with Recorded accompaniment, for which the average perceived synchrony levels remained close to a neutral 3 in a 1-5 scale (a rating of 3 indicates neither disagreeing or agreeing with the perception of synchrony). This suggests that

participants generally did not feel out of synchrony, despite the added challenge of improvising with a recording (see Supplementary Figure S2).

Pairwise comparisons of piano composition style for perceived synchrony showed similar findings as pairwise comparisons of piano composition style for challenge (PC2 of behavioral reports of music playing experience, onto which perceived synchrony loads). Findings for challenge are reported separately in the supplementary results below (see Effects of Piano Composition Style on Behavioral Correlates of Music Playing Experience).

Association between Motor Outcomes

Linear mixed models were used to evaluate the association between motor outcomes using the same methodology as in the main analyses. Three models were tested to investigate the association between each of the motor responses. With total acceleration as the outcome, predictors included one of the other motor responses (mean EMG or drum strike count), the music playing condition (Improvise vs. Beat), accompaniment condition (Live vs. Recorded), and their interaction, as well as control variables for piano composition style, music reward sensitivity (BMRQ), and music training (Gold-MSI), and a random intercept for participant. With mean EMG activation as the outcome, predictors included drum strikes played, the music playing condition (Improvise vs. Beat), accompaniment condition (Live vs. Recorded), and their interaction, as well as control variables for piano composition style, music reward sensitivity (BMRQ), and music training (Gold-MSI), and a random intercept for participant.

For total acceleration as the outcome and drum strike count as a predictor, there was a significant association between total acceleration and the drum strike count (χ^2 (1) = 270.54 , p = <0.00001). There was also a significant main effect of music playing condition (χ^2 (1) = 6.62, p = 0.010) and interaction between music playing condition and accompaniment condition (χ^2 (1) = 6.46, p = 0.011). There were no significant main effects of accompaniment condition (χ^2 (1) = 0.16, p = 0.690), piano composition style (χ^2 (1) = 1.99, p = 0.575), music reward sensitivity (χ^2 (1) = 0.27, p = 0.603), or music training (χ^2 (1) = 0.704, p = 0.401).

For total acceleration as the outcome and mean EMG activation as a predictor, there was a significant association between total acceleration and mean EMG activation (χ^2 (1) = 30.30, p < 0.00001). There was also a significant main effect of piano composition style (χ^2 (1) = 8.78, p = 0.032), while the interaction effect between music playing condition and accompaniment condition approached significance (χ^2 (1) = 3.29, p = 0.070). There were no significant main effects of music playing condition (χ^2 (1) = 0.43, p = 0.514), accompaniment condition (χ^2 (1) = 0.02, p = 0.896), music reward sensitivity (χ^2 (1) = 1.53, p = 0.216), or music training (χ^2 (1) = 0.277, p = 0.599).

For mean EMG activation as the outcome and drum strike count as a predictor, there was not a significant association between mean EMG activation and drum strike count (χ^2 (1) = 1.12, p = 0.289). There was a significant main effect of music playing condition (χ^2 (1) = 14.88, p = 0.0001) and piano composition style (χ^2 (1) = 75.90, p < 0.00001), as well as a significant

interaction between music playing condition and accompaniment condition (χ^2 (1) = 5.83, p = 0.016). There were no main effects of accompaniment condition (χ^2 (1) = 0.15, p = 0.701), music reward sensitivity (χ^2 (1) = 0.07, p = 0.786), or music training (χ^2 (1) = 0.304, p = 0.582).

Despite the association between total acceleration and the other two motor responses, the models continue to demonstrate other significant predictors of total acceleration, showing that the other motor responses do not account for all of the variance in total acceleration.

Effects of Piano Composition Style on Behavioral Correlates of Music Playing Experience

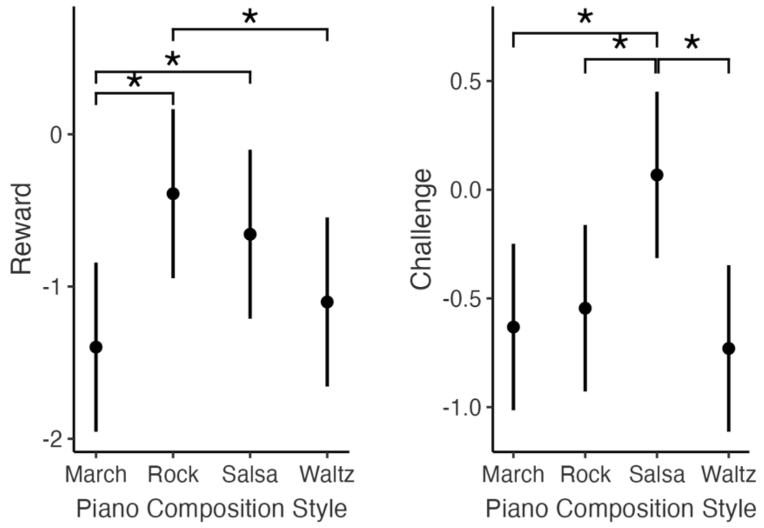

Figure S3: Behavioral Correlates of Piano Composition Style. Reward ratings were lower for the March compared to the Rock and Salsa compositions but not significantly different from the Waltz. Reward ratings were lower for the Waltz compared to the Rock composition. Challenge ratings were highest for the Salsa compared to all other piano compositions. * p < 0.05 after correction for multiple tests.

There was a significant main effect of piano composition style on reported reward (χ^2 (3) = 33.92, p < 0.00001). Pairwise comparisons, corrected for multiple tests, revealed less reward for the March as compared to the Rock (t(444) = -5.33, p < 0.0001) and Salsa (t(444) = -3.92, p = 0.0006) compositions, but not the Waltz (t(444) = -1.57, p = 0.399). Similarly, the Waltz was associated with less reward than the Rock composition (t(444) = -3.76, p = 0.0011) with a trend for less reward compared to the Salsa composition (t(444) = -2.35, p = 0.088). There were no differences in reward for the Salsa and Rock compositions (t(444) = 1.41, p = 0.495).

There was also a significant main effect of piano composition style on reported challenge (χ^2 (3) = 35.42, p < 0.00001). Pairwise comparisons, corrected for multiple tests, revealed greater challenge for the Salsa composition as compared to the March (t(444) = 4.72, p = 0.00002), Waltz (t(444) = 5.39, p = 0.0000007), and Rock (t(444) = 4.14, p = 0.00024) compositions.

Effects of Piano Composition Style on Motor Response to Music Conditions

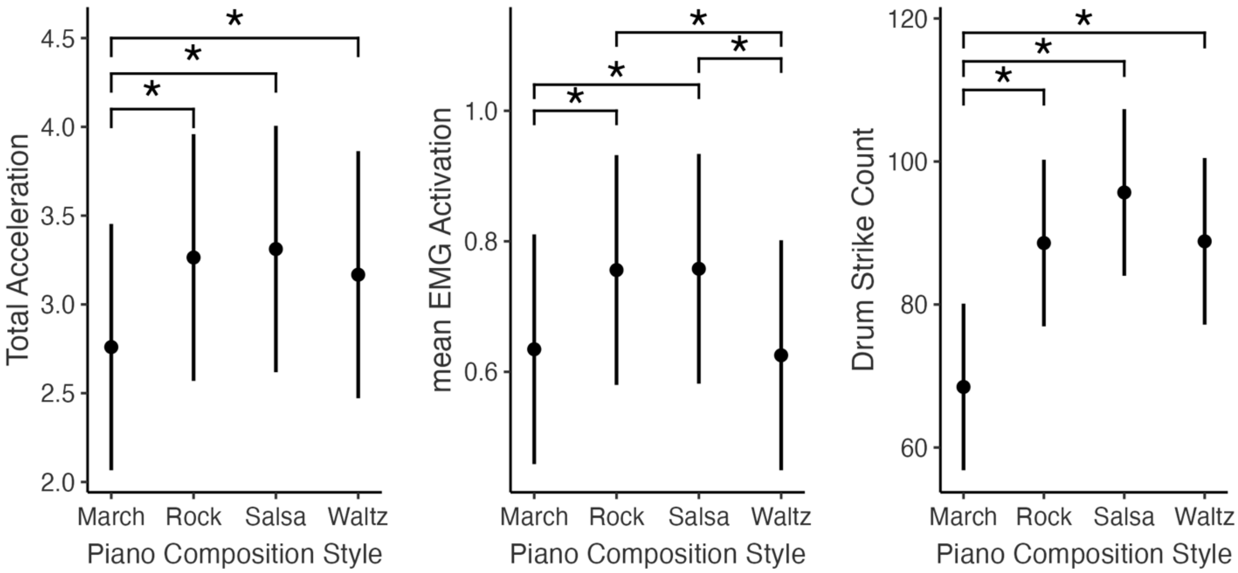

Figure S4: Motor Response to Piano Composition Style. The March was associated with lower total acceleration and drum strike count compared to all other piano compositions. Both the March and Waltz were associated with lower mean EMG activation compared to the Rock and Salsa compositions. *p < 0.05 after correction for multiple comparisons.

There was a significant main effect of piano composition style on total acceleration (χ^2 (3) = 23.99, p = 0.00003). Pairwise comparisons, controlling for multiple tests, revealed lower total acceleration for the March as compared to the Rock (t(432.23) = -3.98, p = 0.0005), Salsa (t(432.00) = -4.41, p = 0.0001, and Waltz (t(432.33) = -3.18, p = 0.0087) compositions. There were no significant differences in total acceleration between the Rock and Salsa (t(432.23) = -0.376, p = 0.982) or Waltz compositions (t(432) = 750, p = 0.877), or between the Salsa and Waltz compositions (t(432.33) = 1.125, p = 0.674).

There was also a significant main effect of piano composition style on EMG activation (χ^2(3) = 82.60, p < 0.00001). Pairwise comparisons, controlling for multiple tests, revealed less EMG activation for the March as compared to the Rock (t(372.08) = -6.16, p < 0.00001) and Salsa (t(372.00) = -6.35, p < 0.00001) compositions, but not the Waltz (t(372.12) = 0.461, p = 0.967). Similarly, there was lower EMG activation for the Waltz as compared to the Rock (t(372.04) = -

6.50, $p < 0.00001$) and Salsa ($t(372.11) = -6.63$, $p < 0.00001$) compositions. There were no significant differences in EMG activation between the Rock and Salsa compositions ($t(372.08) = 0.097$, $p = 0.9997$).

There was also a significant main effect of piano composition style on the number of beats played (χ^2 (3) = 61.13, $p < 0.00001$). Pairwise comparisons, controlling for multiple tests, revealed fewer number of beats played for the March, as compared to the Rock ($t(444) = -5.47$, $p = 0.0000005$), Salsa ($t(444) = -7.39$, $p < 0.0000001$), and Waltz ($t(444) = -5.53$, $p = 0.0000003$) compositions. There were no significant differences in the number of beats played between the Rock and Salsa ($t(444) = -1.92$, $p = 0.220$) or Waltz ($t(444) = -0.066$, $p = 0.9998$) compositions, or between the Salsa and Waltz compositions ($t(444) = 1.86$, $p = 0.249$).

Effects of Piano Composition Style on Autonomic Response to Music Conditions

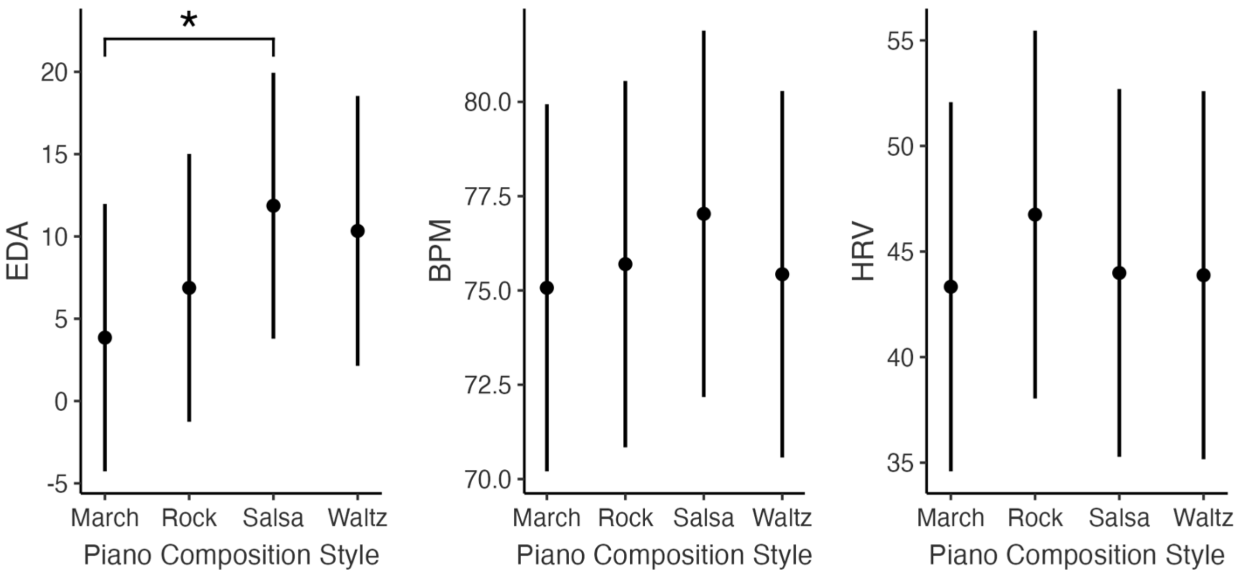

Figure S5: Autonomic Response to Piano Composition Style. The Salsa was associated with higher tonic EDA (indicating greater autonomic arousal) compared to the March. There was no significant effect of piano composition style on BPM or HRV. *$p < 0.05$ after correction for multiple comparisons.

There was a significant main effect of piano composition style on tonic EDA (χ^2 (3) = 8.86, $p = 0.031$), while controlling for the effects of movement by including total acceleration in the model. Pairwise comparisons, controlling for multiple testing, revealed lower tonic EDA for the March as compared to the Salsa composition ($t(345.51) = -2.72$, $p = 0.035$), but not the Rock ($t(347.79) = -1.01$, $p = 0.743$) or Waltz ($t(345.66) = -2.15$, $p = 0.140$) compositions. There were no significant differences between the Rock and Salsa ($t(342.24) = -1.69$, $p = 0.330$) or Waltz

(t(342.16) = -1.15, p = 0.660) compositions, or between the Salsa and Waltz compositions (t(342.82) = 0.51, p = 0.956).

There was no effect of piano composition style on BPM (χ^2 (3) = 7.48, p = 0.058), and no effect of piano composition style on HRV (χ^2(3) = 3.92, p = 0.271).

Effects of Piano Composition Style on Reward with Motor Response as a Predictor

The main effect of piano composition style on reported reward remained significant after adding total acceleration to the model (χ^2(3) = 28.71, p = 0.000003). Similarly, the main effect of piano composition style on reported reward remained significant after adding mean EMG activation to the model (χ^2(3) = 19.90, p = 0.0002), and the main effect of piano composition style on reported reward remained significant after adding the drum strikes played to the model (χ^2(3) = 26.08, p = 0.000009). In each of these three models, the pairwise comparisons of piano composition style on reported reward maintained the same relationships as previously reported.

Supplementary Audio Legends

Supplementary Audio S1: Recording of Salsa style composition used for the Recorded accompaniment conditions for all participants.

Supplementary Audio S2: Recording of Rock style composition used for the Recorded accompaniment conditions for all participants.

Supplementary Audio S3: Recording of Waltz style composition used for the Recorded accompaniment conditions for all participants.

Supplementary Audio S4: Recording of March style composition used for the Recorded accompaniment conditions for all participants.

Supplementary Audio S5: Participant 12 playing with the Rock style composition for the Beat and Live conditions.

Supplementary Audio S6: Participant 12 playing with the Rock style composition for the Beat and Recorded conditions.

Supplementary Audio S7: Participant 12 playing with the Rock style composition for the Improvise and Live conditions.

Supplementary Audio S8: Participant 12 playing with the Rock style composition for the Improvise and Recorded conditions.

Supplementary Audio S9: Participant 13 playing with the Rock style composition for the Beat and Live conditions.

Supplementary Audio S10: Participant 13 playing with the Rock style composition for the Beat and Recorded conditions.

Supplementary Audio S11: Participant 13 playing with the Rock style composition for the Improvise and Live conditions.

Supplementary Audio S12: Participant 13 playing with the Rock style composition for the Improvise and Recorded conditions.
